# Cardiovascular Risk Assessment in Rheumatoid Arthritis: Accelerated Atherosclerosis, New Biomarkers, and the Effects of Biological Therapy

**DOI:** 10.3390/life13020319

**Published:** 2023-01-23

**Authors:** Diana Popescu, Elena Rezus, Minerva Codruta Badescu, Nicoleta Dima, Petronela Nicoleta Seritean Isac, Ioan-Teodor Dragoi, Ciprian Rezus

**Affiliations:** 1 Department of Internal Medicine, “Grigore T. Popa” University of Medicine and Pharmacy, 16 University Street, 700115 Iasi, Romania; 2 Internal Medicine Clinic, “Sf. Spiridon” County Clinical Emergency Hospital, 700111 Iasi, Romania; 3 Department of Rheumatology and Physiotherapy, “Grigore. T. Popa” University of Medicine and Pharmacy, 16 University Street, 700115 Iasi, Romania; 4 Rheumatology Clinic, Clinical Rehabilitation Hospital, 700661 Iasi, Romania

**Keywords:** atherosclerosis, autoimmune disease, rheumatoid arthritis, inflammation, cardiovascular risk, biological therapy

## Abstract

Rheumatoid arthritis (RA), one of the most common of the chronic inflammatory autoimmune diseases (CIADs), is recognized as an independent cardiovascular risk factor. Traditional risk factors such as smoking, arterial hypertension, dyslipidemia, insulin resistance, and obesity are frequently found in RA. Given the increased risk of mortality and morbidity associated with cardiovascular disease (CVD) in RA patients, screening for risk factors is important. Moreover, there is a need to identify potential predictors of subclinical atherosclerosis. Recent studies have shown that markers such as serum homocysteine, asymmetric dimethylarginine, or carotid intima–media thickness (cIMT) are correlated with cardiovascular risk. Although RA presents a cardiovascular risk comparable to that of diabetes, it is not managed as well in terms of acute cardiovascular events. The introduction of biological therapy has opened new perspectives in the understanding of this pathology, confirming the involvement and importance of the inflammatory markers, cytokines, and the immune system. In addition to effects in inducing remission and slowing disease progression, most biologics have demonstrated efficacy in reducing the risk of major cardiovascular events. Some studies have also been conducted in patients without RA, with similar results. However, early detection of atherosclerosis and the use of targeted therapies are the cornerstone for reducing cardiovascular risk in RA patients.

## 1. Introduction

The main cause of death worldwide continues to be CVD. According to the World Health Organization (WHO), CVD causes 17.9 million deaths globally each year, accounting for 32% of all deaths [1,2,3]. Myocardial infarction (MI) and stroke (defining atherosclerotic cardiovascular disease (ASCVD)) are two of the most important complications and contributors to mortality, both of which have atherosclerosis as their underlying process. Moreover, it has been established that these two conditions share a number of risk factors, including traditional cardiovascular risk factors such as smoking, sedentary life leading to overweight or obesity, high blood pressure (BP), glucose intolerance or diabetes, and dyslipidemia [1,2,3,4].

Considering that more than 70% of CVD can be prevented, the research focus has mainly been on modifiable risk factors, in order to identify and treat them, thereby reducing the overall disease burden. Nevertheless, beyond these risk factors, there are several non-traditional factors that have been described as influencing cardiovascular risk. Clinical conditions such as cancer, chronic kidney disease (CKD), infections, chronic obstructive pulmonary disease (COPD), CIAD [4], and hyperhomocysteinemia [5] increase the occurrence of CVD and worsen its prognosis.

Inflammatory rheumatic diseases are chronic conditions involving the joints, muscles, and tissues, causing both joint and systemic manifestations. RA, the most common form of CIAD, is characterized by damage to the synovial membrane [6]. It affects women more frequently than men, with a ratio of 3:1, and is most common after the age of 50. Many studies have focused on the prevalence of the disease, estimating a global prevalence of 0.5–1%, with no significant change between 1990 and 2010 [7,8].

CIAD has been shown to increase cardiovascular risk, with significantly higher rates of cardiovascular mortality and morbidity [4]. According to the 2021 ESC Guidelines on CVD prevention, RA is an independent cardiovascular risk factor, increasing the risk of developing ASCVD by about 50%, even in the subclinical stages or in patients with early-stage RA and symptoms for less than one year [4,9].

Even though in almost half of the cases the increased cardiovascular risk is due to traditional risk factors [10], their management does not reduce it, with RA patients having more than twice the risk of developing MI compared to the general population [11]. This risk is sustained by the characteristics of the disease, such as seropositivity (expressed by the presence of specific antibodies, e.g., rheumatoid factor (RF); anti-citrullinated protein antibodies (ACPAs)), inflammatory syndrome (represented by elevated C-reactive protein (CRP) or erythrocyte sedimentation rate (ESR)), and disease duration or disease activity score (DAS) [12]. On the other hand, systemic inflammation is by itself an important factor in increasing cardiovascular risk, both acutely and over time [13]. Moreover, it appears that RA patients run roughly the same risk of developing acute cardiovascular events as do patients with type 2 diabetes mellitus [14].

Cardiovascular mortality in RA patients is estimated to account for half of all causes, making it the most frequent cause of death [15]. Therefore, in addition to inducing remission or at least reducing disease activity, the goal of therapy in RA is to control chronic inflammation and, thus, reduce cardiovascular risk [16].

With this review, we aim to provide a better understanding of the inflammation–atherosclerosis axis. We consider the common and frequently encountered cardiovascular risk factors that, despite their advanced management, may be difficult to treat in clinical practice. Their relationship with atherosclerosis is examined, as well as the possibility of their use as biomarkers for the detection of early atherosclerosis in RA patients. Moreover, the effects of anti-inflammatory and disease-modifying antirheumatic drugs (DMARDs) (such as biological therapy (bDMARDs)) on cardiovascular risk factors—and especially on the process of atherosclerosis—are reviewed and discussed.

## 2. Cardiovascular Risk Factors in RA and Their Relationship with Atherosclerosis

### 2.1. Arterial Hypertension

Arterial hypertension has been identified as the most prevalent comorbidity in RA patients [17,18,19], which can be explained by several mechanisms, including genetic polymorphism [20], association with other traditional risk factors (e.g., physical inactivity, obesity, alcohol, dyslipidemia, metabolic syndrome) [21], or the use of pathogenic or symptomatic treatments for the disease. Special attention should be paid to patients prescribed corticosteroids, COX-2 inhibitors, or leflunomide, as there are studies showing increased BP in patients taking these therapies. In a recent systematic review, Hadwen et al. [22] demonstrated that corticosteroids and COX-2 inhibitors may increase the risk of arterial hypertension in RA patients. Among the synthetic DMARDs used in managing RA, leflunomide is the one that carries the greatest risk of inducing hypertension, so other drugs should be used first in the presence of this risk factor. In contrast, methotrexate (MTX) seems to have protective effects, since it has been negatively associated with arterial hypertension in several studies [23,24,25].

There is evidence showing the relationship between inflammation (as illustrated by increased levels of CRP or inflammatory cytokines such as interleukin (IL)-6) and high BP [26,27]. Arterial hypertension is associated with accelerated atherosclerosis in RA patients, which is mainly based on their pro-inflammatory status, with studies showing that hypertensive RA patients have a higher risk of acute cardiovascular events (such as MIs) than patients with normal BP [23,28]. On the other hand, a study published by Yu et al. [29] showed an inverse U-shaped relationship between CRP and systolic BP (SBP) in both RA and non-RA patients, meaning that an increased CRP level (i.e., ≥6 mg/L) led to decreased SBP. The mechanisms mediating these changes are not fully understood, which is why new horizons are open for future studies to clarify the link between inflammation and BP dysregulation.

### 2.2. Dyslipidemia

Dyslipidemia, defined as a change in serum lipid concentration, is an important modifiable cardiovascular risk factor in the general population, given its close links with atherosclerosis [30]. Increased cardiovascular risk due to dyslipidemia in RA patients is a result of several factors, including disease activity, specific inflammatory processes, sedentary lifestyle and, last but not least, the so-called “lipid paradox”. According to London et al. [31], disease activity is negatively correlated with cholesterol levels. Since then, numerous studies have focused on this complex relationship between dyslipidemia and cardiovascular risk in RA patients, with results that, although incompletely understood, are still valid today.

The most frequently reported changes in the lipid profile are quantitative. The activity of the disease, expressed by the CRP, is correlated with the inflammatory status, resulting in increased cholesterol consumption and a reduction in its synthesis. Thus, low levels of total cholesterol (TC) and low-density lipoprotein cholesterol (LDL-C), along with high levels of high-density lipoprotein cholesterol (HDL-C), were observed in RA patients with active disease [23,32,33]. Paradoxically, in these patients, although TC levels and those of its fractions remain low, the cardiovascular risk persists, most likely due to the inflammatory status, but also to changes in the structure and functions of lipoproteins [32,34]. These observations have been referred to as the “lipid paradox”, and a U-shaped relationship has been developed [21,35].

In non-RA patients, among other important roles (e.g., transport of cholesterol from extrahepatic tissues to the liver, the site of its catabolism; inhibition of monocyte adhesion; antithrombotic properties), HDL-C is thought to confer protection against oxidized LDL-C (oxLDL-C) (responsible for the development of atherosclerotic plaques) via paraoxonase-1. On the other hand, in RA patients, these properties of HDL-C are no longer observed. Instead, it has been observed that HDL-C’s ability to inhibit oxLDL was affected by inflammation, with paraoxonase-1 being greatly reduced in these patients [36]. These qualitative changes in the lipid profile of RA patients lead to increased cardiovascular risk through the development and progression of atherosclerosis.

### 2.3. Obesity and Insulin Resistance

A number of chronic diseases have been linked to obesity, which is a major health problem in the modern world. Given that obesity leads to CVD independently of other risk factors [37], attention has been focused on the impact of obesity associated with RA on cardiovascular mortality. It is well known that adipokines (Table 1)—cytokines secreted in excess by adipose tissue—are involved in the development of inflammation and insulin resistance, thereby initiating and promoting atherosclerosis. As hormones that are closely related to the immune system and various organs (e.g., heart, brain, liver), adipokines induce endothelial dysfunction with platelet activation and secondary pro-thrombotic, pro-atherogenic, and systemic pro-inflammatory effects [38].

The effects of obesity on RA have been intensively studied over time [39,40], with one of the earliest and largest studies being published by Lu et al. (Nurses’ Health Survey/Nurses’ Health Survey II) [39]. This study established a positive, age-dependent relationship between overweight/obesity and the occurrence of RA. As in the case of dyslipidemia, the relationship between body mass index (BMI) and the risk of cardiovascular or all-cause mortality was inverse in RA patients compared to non-RA patients. In summary, it has been observed that overweight or obese RA patients have a lower relative risk of cardiovascular death than normal-weight patients. This effect has been called “the obesity paradox in RA” and has gained the attention of subsequent studies [41]. It was later shown that BMI is not an accurate predictor of mortality among RA patients, mainly due to accelerated catabolism in patients with active autoimmune disease leading to unintentional weight loss [42,43].

**Table 1:** Adipokines and their effects in RA patients.

Adipokine	Functions	Source	Effects in RA Patients	Reference
**Adiponectin**	*Anti-inflammatory effect* *Anti-atherogenic effect*	Adipocytes	Pro-inflammatory effect Correlated with disease activity, disease progression, and inflammatory markers	[44,45]
**Leptin**	Pro-inflammatory effect Appetite and weight regulator	Adipocytes	Pro-inflammatory effect Correlated with disease activity and progression, as well as with IL-6 levels	[44,46,47]
**Visfatin**	Pro-inflammatory effect Promotion of B-cell differentiation	Adipose tissue, liver, bone marrow, muscle	Pro-inflammatory effect Correlated with inflammatory markers and disease activity Expression of visfatin seems to be linked to decreased cardiometabolic risk	[44,48,49]
**Resistin**	Pro-inflammatory effect Promotion of immune cell recruitment and immune cell activation	Macrophages, adipocytes	Pro-inflammatory effect Systemic levels may depend on RA disease duration or severity Synovial levels seem to be correlated with inflammatory markers and disease activity	[44,50]
**Omentin**	*Anti-inflammatory effect* *Anti-atherogenic effect*	Stromal vascular cells, adipocytes	Systemic levels were associated with inflammatory markers, while tissue concentrations were neutral	[44,51]
**Progranulin**	*Anti-inflammatory effect* (by competitive binding to tumor necrosis factor (TNF))	Adipocytes, macrophages, chondrocytes	Pro-inflammatory marker Correlated with disease activity and progression Is a key player in the preservation of cartilage integrity	[44,46,52]

### 2.4. Homocysteine

Homocysteine, a sulfhydryl-containing amino acid, is recognized as an independent cardiovascular risk factor. Hyperhomocysteinemia can lead to atherosclerosis by several different mechanisms. One of them is through oxidative stress, which causes nitric oxide (NO) depletion with endothelial dysfunction and atherothrombosis, but also contributes to the formation of oxLDL with the release of pro-inflammatory cytokines by spumous cells. Moreover, hyperhomocysteinemia, through asymmetric dimethylarginine (ADMA)—which is derived from S-adenosyl methionine (an intermediate in the metabolism of homocysteine)—can stimulate the proliferation of the arterial wall’s smooth cells [53,54,55]. These alterations in subclinical arterial structure and function contribute to atherosclerotic plaque formation and vascular calcification. Recently, Karger et al. [5], based on the Multi-Ethnic Study of Atherosclerosis (MESA) cohort, confirmed the results of previous studies on the relationship between increased homocysteine levels and the prevalence of vascular calcification.

The link between inflammatory status in RA patients and hyperhomocysteinemia has been demonstrated in several studies over time. For example, in 1997, Roubenoff et al. [56] showed that homocysteine levels were about 33% higher in RA patients than in the control group, assuming that this could be an explanation for the increased cardiovascular mortality seen in these patients. Later, Tekaya et al. [57] found that homocysteine levels were associated with high disease activity, CRP, age, and male gender. In another cross-sectional study using the Kyoto University Rheumatoid Arthritis Management Alliance (KURAMA) database, Katsushima et al. [58] demonstrated that hyperhomocysteinemia was strongly and positively correlated with DAS-28-ESR but, more importantly, this relationship was stronger in the non-remission group than in the remission group. Regarding the impact of DMARDs on homocysteine levels in RA patients, glucocorticoid therapy is linked to a quick and sustained reduction in plasma homocysteine concentrations, which may have an effect on cardiovascular risk [59]. In contrast, treatment with MTX alone or in combination with sulfasalazine (SSZ) resulted in a persistent increase in plasma homocysteine, which is why therapy in these patients should be adjusted by adding folic acid (5 mg/week) to reduce homocysteine levels [60].

## 3. Inflammation and Atherosclerosis

Rudolf Virchow, the father of cellular pathology, was the first to describe the pathophysiological mechanism of thrombosis, later synthesizing, in Virchow’s triad, the risk factors that predispose to thrombus formation. Moreover, in 1856, in one of his publications, he stated the inflammatory character of atherosclerotic plaques as follows: “in some particularly violent cases of softening manifests itself even in the arteries not as the consequence of a real fatty process, but as a direct product of inflammation” [61]. Later, in 1999, Russel Rose hypothesized that atherosclerosis is an inflammatory disease and that atheromatous plaque formation is sustained by an important immunological component [62].

**Figure 1:**
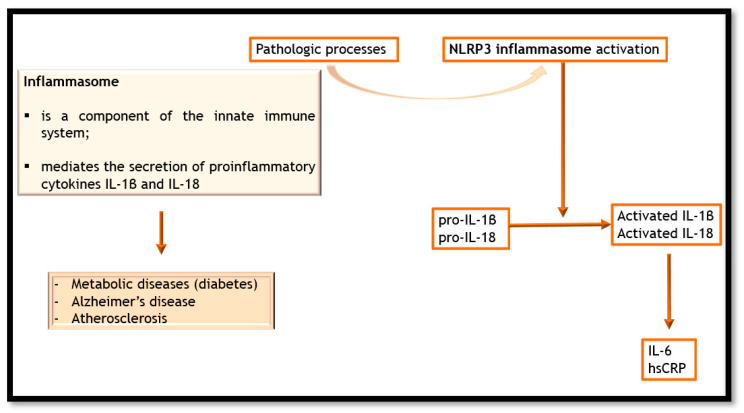
Inflammasome activation and its effects. hsCRP- high-sensitivity CRP; NLPR3 inflammasome contributes to the regulation of innate immune system and controls the release of pro-inflammatory cytokines.

Atherosclerotic plaque formation begins with endothelial dysfunction and endothelial cells (ECs) undergoing inflammatory activation. ECs play an important role in the pathogenesis of atherosclerosis through their barrier capacity and paracrine/endocrine secretory functions. They regulate vasodilatation, monocyte infiltration, and platelet aggregation via vasoactive mediators such as endothelin-1 (ET-1), NO, prostacyclin, angiotensin II (Ang II), vascular cell adhesion molecule 1 (VCAM-1), and intercellular adhesion molecule 1 (ICAM-1), as well as vascular endothelial growth factor (VEGF) and platelet-derived growth factor (PDGF). Leukocytes and monocytes enter the subendothelial space and secrete chemokines and other chemoattractant molecules. Monocytes become tissue macrophages, which internalize lipoprotein particles and generate foam cells. These cause the secretion of inflammatory cytokines, reactive oxygen species (ROS), and other mediators. After subendothelial accumulation, LDL becomes oxLDL; moreover, cholesterol accumulation leads to inflammasome activation, which results in the cleavage of IL-1β into its biologically active form (Figure 1) [63,64,65,66]. Macrophages are classified into two categories: M1, secreting pro-inflammatory factors that participate in tissue damage; and M2, secreting anti-inflammatory factors. Under homeostatic conditions, macrophages have atheroprotective effects, but under pathological conditions they are involved in the local immune response [63,67]. Vascular smooth muscle cells (VSMCs) can change their phenotypic form and secrete cytokines (e.g., IL-6, IL-8, and monocyte chemoattractant protein-1 (MCP-1)) and a large number of extracellular matrix (ECM) proteins, such as elastic fibers, collagens, proteoglycans, and matrix metalloproteinases (MMPs) [68]. Depending on their phenotype, VSMCs can have a pro-inflammatory or anti-inflammatory influence. A macrophage-like phenotype, a mesenchymal stem cell (MSC)-like phenotype, a fibromyocyte phenotype [69], an osteogenic phenotype [70], an EC-like phenotype [71], an adipocyte-like phenotype, and an intermediate cell phenotype [72] can be found in the pro-inflammatory model.

The inflammatory response triggered by endothelial dysfunction and the dysregulation in lipid metabolism is followed by an adaptive immune response, involving T and B cells.

Through their activation and secretion of cytokines, T-helper (Th) 1 cells contribute to atherogenesis. Th2 cells have both pro- and anti-atherosclerotic properties. These two types of T lymphocyte (LT) interact with B cells and IL secretion [73]. Regulatory T cells (Tregs) inhibit Th activity while promoting the anti-inflammatory phenotype of macrophages [73]. A reduced number of Tregs in atheromatous plaques is representative of the local inflammation that occurs there [74]. Recently, researchers have demonstrated that ApoB-reactive T cells evolve from Tregs that have lost their atheroprotective effects [75]. Among symptomatic patients and those with recent cardiovascular events, CD4-positive and CD8-positive T cells are activated and differentiated [76]. B lymphocytes (LB) induce inflammation due to antibody production, but mainly due to the secretion of pro-inflammatory factors. B1 and B2 cells are most commonly present in atherosclerotic plaques. An important role of the two types of B cells is the secretion of IL-10, which has a repressive impact on inflammation. In atherosclerosis, the plaque number of IL-10 is small, and this helps to promote inflammation. Anti-LB medication appears to have beneficial effects in patients with atherosclerosis, demonstrating B-cell involvement [63].

Taking into account the inflammatory component present at the atherosclerotic level, several studies have been carried out to investigate the influence of anti-inflammatory medication on the atherosclerotic process.

The LoDoCo (Low-dose colchicine for secondary prevention of cardiovascular disease) trial examined the effects of colchicine in patients with CVD, looking at the eventual reduction in the risk of cardiovascular events. A dose of 0.5 mg/day was used versus a placebo in 532 patients with stable coronary artery disease after 3 years of follow-up. The results were positive, with the combination of colchicine, high-dose statin, and another standard secondary prevention therapy preventing recurrent cardiovascular events in these participants [77]. This was followed by LoDoCo 2—a randomized, controlled, double-blind trial that involved 5522 patients with chronic coronary disease. A dose of 0.5 mg of colchicine was used in 2762 of the patients, with the other 2760 being the placebo group. The follow-up period was at least 12 months. At the end of follow-up, patients who received colchicine had a lower risk of cardiovascular events compared to the placebo group [78].

Considering the positive effect that MTX has on inflammatory biomarkers and cardiovascular risk in patients with rheumatic diseases, the CIRT (Cardiovascular Inflammation Reduction Trial) study was developed. This was a randomized, double-blind trial in which 4786 patients with a history of MI or multivascular coronary disease along with type 2 diabetes or metabolic syndrome were included. The dose of MTX was 15–20 mg/week. The trial was stopped after a median follow-up of 2.3 years. The results showed that MTX did not lead to a decrease in cardiovascular events or inflammatory markers. Moreover, there were increases in liver enzymes, decreases in the associated number of leukocytes and hematocrit, and an increase in the incidence of non-basal-cell skin cancers compared to the placebo group [79].

The involvement of cytokines in the formation of atherosclerotic plaques has become a possible treatment target over time. IL-1β is one of the most important cytokines involved in pathogenesis; therefore, targeted therapies towards it could have positive effects. The CANTOS (Canakinumab Anti-Inflammatory Thrombosis Outcome Study) trial evaluated the medical effects of anti-IL-1β therapy (canakinumab) through a randomized double-blind trial. A total of 10,061 patients with previous MI and a high-sensitivity CRP (hs-CRP) level > 2 mg/dL were included. Three different doses of canakinumab (50 mg, 150 mg, and 300 mg administered every 3 months subcutaneously) were compared with a placebo. The results showed a decrease in CRP in CANA patients. After 48 months, the average reduction in those on 50, 150, and 300 mg was 26%, 37%, and 41%, respectively. Lipid levels were not influenced by the initial value. A reduction in cardiovascular events was also demonstrated in patients from the CANA group. Regarding adverse effects, an increase in mortality due to infections or sepsis was observed. Thus, CANTOS certified inflammatory involvement in atherosclerosis [80]. Another targeted cytokine is IL-6. The ASSAIL-MI (Assessing the effect of anti-IL-6 treatment in MI) trial, which is currently in phase II, has highlighted that tocilizumab (TCZ) improves outcomes in patients presenting with an acute ST segment elevation MI (STEMI), due to reduced myocardial damage [81]. Moreover, in STEMI patients, IL-6 inhibition induced a decrease in neutrophil numbers and appeared to reduce neutrophil function, which may be connected to TCZ’s favorable effects on myocardial salvage [82].

Increased cardiovascular risk due to accelerated atherosclerosis and its relationship with inflammation in RA patients are well proven [83]. In addition to the fact that CVD and RA share common risk factors—such as genetic (e.g., genetic polymorphism) and environmental factors (e.g., smoking, obesity, metabolic syndrome)—the increased levels of inflammation in these patients make the atherosclerotic plaques unstable and prone to rupture.

A wide range of disease-associated single-nucleotide polymorphisms (SNPs) are shared by RA and CVD. Human leukocyte antigens (HLAs) are involved in the pathogenesis of RA, and HLA-DRB1*04 is considered to be a risk factor for both RA and CVD [84]. Moreover, different genetic forms of inflammatory mediators appear to be common risk factors for RA and CVD. Two variants of TNF-α are known to be risk factors involved in complications of both RA and CV. From the IL-1 family, IL-33 is responsible for mediating CV events in RA patients [85]. On the other hand, interferon (IFN) does not seem to be involved in CVD in RA patients [86]. IL-6 is another IL that is involved in both RA and CVD due to atherosclerosis. Increased levels of IL-6 may predict the risk of MI, as revealed by a prospective study that included nearly 15,000 apparently healthy patients [87]. Moreover, IL-6 can be considered to be a negative prognostic factor for acute coronary events [88].

Citrullination is a process that is part of RA’s pathogenesis and has been a point of interest in recent years. ACPAs are essential for diagnosis and are used to monitor disease activity. The presence of ACPAs and RF are markers of ischemic heart disease [89]. Citrullination processes were highlighted in the myocardial interstitium of patients and in the atheromatous plaques of RA patients [90]. In this way, ACPAs are determinant of the atherogenesis process [91]. Moreover, according to the MESA cohort and the Northwick Park Heart Study, ACPAs can also cause CVD in patients without RA [92].

Although not as well-researched, antibodies against carbamylated proteins can appear in RA patients and can be detected even before clinical diagnosis [93]. Under these conditions, the atherosclerosis process can be promoted by carbamylated HDL-C and LDL-C. Carbamylated HDL-C influences the absorption and efflux process of cholesterol towards macrophages [94]. On the other hand, carbamylated LDL-C promotes atherogenesis by altering the endothelium, the proliferation of smooth muscle cells, and the stimulation of monocytes’ adhesion to altered ECs [95]. Therefore, these antibodies are associated with subclinical atherosclerosis in RA patients [96]. It is important to mention that these antibodies are also associated with an increase in cardiovascular risk in other diseases [97].

## 4. Assessing Cardiovascular Risk in RA

### 4.1. Biomarkers Predictive of Cardiovascular Risk in RA

#### 4.1.1. Lipid Profile

Given the paradox of low LDL-C levels in RA patients, a question has been raised as to whether changes in lipid profiles over time can predict cardiovascular risk in these patients. Myasoedova et al. published a retrospective cohort study in which they looked at the relationships between lipid levels, inflammatory status in RA, and cardiovascular risk. This study confirmed the positive link between increased inflammatory status, low TC levels, and increased cardiovascular mortality. As explained above, the relationship between TC levels and cardiovascular risk was not linear but, rather, represented in the form of a “U-shaped curve” [32]. These particularities have been highlighted in other subsequent studies [33,98].

Recently, Giles et al. published a study based on four cohorts of CVD. Excluding patients undergoing hypolipemiant therapy, they compared coronary artery calcium (CAC) scores in RA patients versus non-RA patients with LDL-C plasma levels. Their results strengthen the idea of an increased cardiovascular risk for RA patients with very low LDL-C levels (defined as an LDL-C < 70 mg/dL), three-quarters of whom had a CAC score ≥ 100 units (this elevated CAC score was associated with the occurrence of cardiovascular events). They also correlated these changes with other risk factors, such as white race, history of smoking, and normoponderal status [99].

#### 4.1.2. Homocysteinemia and ADMA

Several previous studies have tried to determine whether homocysteine can serve as a potential predictive cardiovascular risk factor. For example, a prospective cohort study enrolling patients with no history of acute cardiovascular events (i.e., stroke or MI) demonstrated a link between elevated serum homocysteine values and increased risk of both cardiovascular events and death [100]. Furthermore, another study showed that hyperhomocysteinemia was more common in younger patients (<56 years old) who experienced more than one acute cardiovascular event in evolution (such as stroke, MI, or death) than in those who did not. An important conclusion of this study was that high homocysteine levels at admission may serve as a potential predictor for worse late cardiac events in patients who have premature atherosclerotic diseases [101]. This position was reinforced by another recent study that highlighted the potential predictive role of homocysteine for increased major adverse cardiovascular events (+) in female patients [102].

The relationship between hyperhomocysteinemia and cardiovascular risk in RA patients has also been studied in several works [103]. Balkarli et al. [104] found that inflammatory mediators such as IL-6 and TNF-α, along with homocysteine, are simultaneously increased in RA patients, which may lead to the development of ASCVD.

Due to its pathophysiological involvement in endothelial dysfunction, ADMA has attracted attention in recent years for its potential role as a biomarker of subclinical atherosclerosis. A direct relationship between ADMA levels and acute cardiovascular events, such as stroke or ASCVD, was recently shown in a meta-analysis of 22 cohort studies enrolling over 20,000 patients [103]. In addition, a correlation between ADMA and flow-mediated dilation (FMD) or cIMT has been described, the latter being considered to be a parameter for the detection of subclinical atherosclerocsis [103,105].

Regarding the link between ADMA levels and markers of subclinical atherosclerosis in RA patients, there are some data showing a positive correlation. The greatest impact is in patients with high disease activity, in whom a significant correlation has been demonstrated between ADMA levels and the cIMT. Positive associations have also been described between ADMA levels and CRP and DAS-28, as well as between ADMA levels and ACPA titers—particularly in the early stages of rheumatic disease—or between ADMA levels and homocysteine levels [106,107,108]. In conclusion, ADMA may be a good predictor of subclinical atherosclerosis in RA patients, but further studies are needed to strengthen this position, as well as to identify targeted therapies to reduce cardiovascular risk.

#### 4.1.3. MicroRNAs

MicroRNAs (miRNAs) are a class of small, single-stranded, non-coding ribonucleic acids (RNAs) between 18 and 25 nucleotides in length. Their genesis occurs initially in the nucleus by transcription from the DNA molecule, resulting in pri-miRNAs. Furthermore, they are recognized by an enzyme–protein complex and cleaved into precursors that mature at the cytoplasmic level. The role of mature miRNAs is to regulate post-transcriptional gene expression [109].

MiRNAs are known to be involved in the physiopathological complex process of atherosclerosis [110]. Different miRNAs—such as miR-126, miR-31, miR-17-3p, miR-146, or miR-223—cause endothelial dysfunction through a series of pathological processes such as the adhesion of molecules such as ICAM-1, E-selectin, or VCAM-1, with the recruitment of new white cells or inhibition of NO release leading to atherosclerotic plaque formation and/or destabilization. Moreover, given the links between dyslipidemia, inflammation, and atherosclerosis, several miRNAs (i.e., miR-27a/b, miR-146a, miR-125a-5p, miR-155, miR-301b, miR-302a) have demonstrated their role in lipid metabolism. They seem to be involved in the absorption, esterification, and efflux of cholesterol, as well as in the process of foam cell development, reducing their number [111,112,113].

In addition, miRNAs are also involved in other cellular processes, such as development, proliferation, invasion, cell survival, and apoptosis. Moreover, due to their capacity to influence adaptive responses and the differentiation of B and T cells, as well as lipid uptake and efflux or cytokine synthesis, they have been described in some autoimmune diseases [114]. In RA patients, some miRNAs (e.g., miR-22, miR-38, miR-146, miR-48) have been associated with an increased risk of the development and progression of this autoimmune disease [115], with several studies showing their involvement in patients in the early stages of the disease, but especially in those who eventually developed RA [116,117]. Furthermore, Renman et al. have demonstrated that some miRNAs have a modified expression not only in the sera of RA patients, but also in their first-degree relatives [116]. A recent meta-analysis illustrated the relationship between miRNAs and disease activity by directly and positively correlating miR-146a with DAS-28-ESR [117]. Other studies have found a link between MiR-22 or miR-125b and DAS-28, CRP, and ESR [118], as well as between miR-451 and DAS-28, CRP, and IL-6 [119].

The link between miRNAs and CVD in RA patients has also been studied over time. For example, in a recent study, Taverner et al. demonstrated that decreased expression of miR-425-5p in men was related to a higher risk of subclinical arteriosclerosis, while miR-451 in women was related to lower levels of subclinical arteriosclerosis and lower arterial stiffness in the entire RA cohort. In this study, miRNA levels were directly related to the measurement of cIMT using ultrasound [120]. Expressions of miR-425-5p and miR-451 were also assessed prior to this study, showing similar plasma levels between patients with RA and acute MI, but different from the control group [121]. Another studyhighlighted the link between miRNA expressions and Agatston score—a score for CAC. The results led to the formulation of a list of miRNAs that have high predictability for coronary atherosclerosis in RA patients [122].

Taken together, miRNAs could be used as biomarkers of CVD in RA patients, mainly because they possess great stability in plasma. Future studies are needed to create a panel of miRNAs that can be used as predictors of ASCVD in RA patients.

#### 4.1.4. Anti-β2-Glycoprotein-1 (anti-β2GPI) IgA Antibodies

The involvement of the immune system through its activation and synthesis of pro-inflammatory markers in the development, progression, and destabilization of atherosclerotic plaques is well known. β2GPI is a single-chain polypeptide amino-acid residue compound, known as the primary antigenic target for antibodies involved in thromboembolic complications, and commonly found in patients with CIAD [123]. It has also been found that β2GPI is co-located with CD4-positive lymphocytes and with oxLDL, with the latter forming the *oxLDL/β2GPI* complex by binding oxLDL to the polypeptide, which is not common for native LDL [123,124]. This complex has major implications in the process of accelerated atherosclerosis and is found at increased levels in patients with CIAD, as well as with non-autoimmune diseases such as type 2 diabetes mellitus [125] or CKD [126]. Additionally, the risk of acute MI, unstable angina, or stroke was independently correlated with anti-β2GPI IgA antibodies [127].

The role of anti-β2GPI IgA antibodies in atherosclerotic plaque progression and cardiovascular risk in RA patients is not well studied compared to antiphospholipid syndrome. One study showed a positive correlation between anti-β2GPI IgA and cIMT [128], unconfirmed by others [129,130], but without a direct relationship with the presence or progression of atheromatous plaques. New horizons have been opened with the publication of a recent study that enrolled 150 participants who were subjected to coronary computed tomography angiography for plaque evaluation, with promising results. This is the only study to date that has shown a clear link between anti-β2GPI IgA and the progression of atherosclerotic plaques or their transition to extensive or obstructive ones [131].

### 4.2. Predictive Imaging Markers of Cardiovascular Risk in RA

#### 4.2.1. cIMT

IMT is an easy index to assess using B-mode Doppler ultrasonography of the carotid arteries. The investigation has the advantages of being accessible, replicable, non-invasive, quick, and easy to perform, without requiring special preparation in advance. This is extremely useful for screening patients at high cardiovascular risk, as elevated cIMT (≥1 mm) is correlated with the risk of acute cardiovascular events such as stroke or MI [13,132]. Studies have demonstrated strong predictive value for cardio- and cerebrovascular complications, as well as close links with most traditional cardiovascular risk factors, inflammatory syndrome, and hyperhomocysteinemia [13,133,134,135]. Doppler ultrasound of the carotid arteries also highlights the presence of atheromatous plaques, as well as the degree of stenosis that they produce.

In order to identify patients predisposed to the development of acute cardiovascular complications, several studies have evaluated the importance of imaging plaque identification and calculating the cIMT in RA patients. The findings showed that RA patients have higher cIMT and more frequent carotid atherosclerotic plaques than patients without RA [136,137,138]. Some studies have demonstrated an association between cIMT and inflammatory markers, such as ESR, CRP, or IL-6 [139,140], while others have reported a relationship with ACPA seropositivity [141]. As expected, a link between cIMT and DAS-28 was also detected by Ambrosino et al. [142]. Similar results have also been identified by Wah-Suarez et al. [143], as well as by Gonzales Mazario et al. [144] who, additionally, correlated cIMT with disease duration. Furthermore, it has been demonstrated that RA patients with carotid atherosclerotic plaques sustain a higher risk of acute cardiovascular events and cardiovascular mortality. One of the most noteworthy things is that carotid plaques have a greater ability to predict cardiovascular risk than the modified European Alliance of Associations for Rheumatology (EULAR) systematic coronary risk evaluation (mSCORE) [145]. A positive correlation between cIMT and traditional cardiovascular risk factors has been reported, and the results of that study suggested that the evaluation of cIMT as a cardiovascular risk predictor can be used for RA patients with low CVD [146].

#### 4.2.2. CAC Scores

Determination of CAC score is a method for the diagnosis and evaluation of subclinical atherosclerosis, especially in asymptomatic patients, since it has diagnostic value even before the signs and symptoms of myocardial ischemia appear. Using multidetector cardiac computed tomography (CT), the score is calculated based on the most widely used algorithm—the Agatston score. Although the investigation (CT) does not require a contrast substance, a minor disadvantage is the low irradiation dose. The CAC score quantifies the extent and density of calcium deposits in relation to the examined area, making it useful not only in diagnosis, but also in stratifying the risk and the severity of atherosclerosis [147].

Evaluation of CAC score has been endorsed by the American and European cardiology associations to improve cardiovascular risk stratification and assessment. Since there are data showing a link between Framingham risk score and the CAC score in RA patients, studies have been conducted to determine whether the CAC score might be a good predictor of subclinical atherosclerosis in RA patients [147,148]. As previously shown, elevated CAC score was associated with very low LDL-C levels, demonstrating positive predictive value for the occurrence of acute cardiovascular events [99]. The study carried out by Karpouzas et al. [149] had significant results. RA patients had higher CAC, a greater number of plaques—especially for the more vulnerable types, such as non-calcified and mixed ones—the most prevalent multivessel disease and, most importantly, increased risk of mild-to-moderate and severe plaque burden compared with controls. Furthermore, a recent study [149] examined the link between various cardiovascular risk scores used in RA patients and CAC score, with the Expanded Risk Score in Rheumatoid Arthritis (ERS-RA) showing the highest correlation coefficient.

Thus, studies have confirmed that CAC score can be used as an indirect marker of atherosclerotic burden in both RA and non-RA patients.

## 5. The Effects of Biological Therapy on Cardiovascular Risk Factors in RA

Therapies used in RA include DMARDs, categorized into conventional synthetic DMARDs (csDMARDs), targeted synthetic DMARDS (tsDMARDs), and bDMARDs. csDMARDs are used as first-line therapy in the absence of contraindications, with MTX being the first option. Moreover, when disease activity is high, low doses of glucocorticoids can be used for a short period of time as a bridging therapy. If after 3 months of proper therapy there is no improvement in the disease, or if after 6 months there is no therapeutic target (defined as remission or low disease activity), either the csDMARD is replaced by a different one or a second one is added. Failure of two csDMARDs at maximum tolerated doses for at least 3 months, presence of inflammatory syndrome, and/or very high disease activity are indications for bDMARDs or tsDMARDs [150,151,152].

The introduction of biological therapies (defined as biotechnologically derived therapeutic agents that modulate inflammation and the immune system and act against cytokines, tissue receptors, or co-stimulatory molecules; they have specific action by binding only to the molecule against which they were synthesized) has greatly improved the prognosis of RA patients. bDMARDs have demonstrated their beneficial effects by achieving therapeutic targets (e.g., inducing disease remission, slowing disease progression), improving quality of life, and reducing signs and symptoms of the disease [152,153,154,155]. With the improvement of preventive and therapeutic measures, the life expectancy of patients with CIAD has increased considerably, but so have the mortality and disability rates due to atherosclerotic vascular events. Understanding and updating knowledge about the pathophysiological mechanisms of biological therapy has led to the hypothesis that it can reduce cardiovascular risk by improving inflammation and, thus, slowing the progression of atherosclerosis. As shown above, the CANTOS and ASSAIL-IM trials have demonstrated the positive impact of molecules targeting inflammatory cytokines on the evolution of patients with acute cardiovascular events [80,81,82].

Although widely used, bDMARDs have the disadvantage of high cost, making them less accessible in low-income countries. Based on this aspect, new products with the same antigenic determinants have been developed in recent years. They are called biosimilars and appear to have much the same therapeutic effects as the original molecules. Moreover, they have been demonstrated to have efficacy and safety profiles that are similar to those of the original bDMARDs [156,157].

The main biological molecules used in RA patients, along with their mechanisms of action, are summarized in Table 2.

### 5.1. Anti-IL-6

IL-6 is a cytokine with pleiotropic effects in inflammation, modulation of immune responses, regenerative processes, hematopoiesis, and metabolism. Synthesized from the initial stage at the site of inflammation by several cell types—such as macrophages, adipocytes, ECs, or smooth muscle cells—IL-6 causes the release of acute-phase reactants from the liver, such as CRP, fibrinogen, haptoglobin, and serum amyloid A (SAA). It is important to note that the transition from the acute to the chronic phase of inflammation is made by the recruitment of the leukocyte infiltrates, while neutrophils are transformed into monocytes or macrophages. In this stage, an important role is played by the soluble IL-6 receptor α (sIL-6Rα) [158,159]. Its important role in the acute and chronic phases of inflammation has made this particular cytokine a key player in the development and progression of atherosclerosis. There are studies that have demonstrated the role of IL-6 as a risk factor for coronary atherosclerosis. For example, Saremi et al. [160] showed an association between IL-6 values and CAC, independent of other cardiovascular risk factors. Another study [87] highlighted a link between increased IL-6 levels and MI risk. Studies in this direction have laid the groundwork for the hypothesis that IL-6 could be a therapeutic target for atherosclerosis.

The first anti-IL-6 drug approved for the treatment of RA, TCZ, has been investigated in several studies. Clear data showing changes in lipid profiles—specifically in the serum lipid levels—have raised concerns about increased cardiovascular risk secondary to the dyslipidemic process. The MEASURE study [161] showed that adding TCZ to MTX increased TC, LDL-C, and triglycerides more than MTX alone; in addition, another report compared monotherapy with TCZ with monotherapy with MTX, with the results favoring MTX in terms of lipid profile [162]. In another study, TCZ alone resulted in greater increases in TC and LDL-C than the combination of two csDMARDs (MTX plus hydroxychloroquine) [163]; meanwhile, in the ADACTA study, comparing TCZ with another bDMARD, TCZ had a more pronounced impact on serum lipid levels than adalimumab [164]. Further analysis concluded that although these changes in lipid profile occurred, long-term use of TCZ reduced the cardiovascular risk due to atherosclerosis. The explanation was found in the same studies, which showed that although TCZ had a negative impact on serum lipid levels, its effects on lipid function and quality were beneficial. Therefore, it was observed that the increase in serum lipids led to an improvement in inflammation, with a reduction in inflammatory markers such as fibrinogen, D-dimer, phospholipase A2, and SAA [161,163,164]. This makes the lipid changes more anti-atherogenic than pro-atherogenic [165]. Furthermore, using TCZ was linked to lower lipoprotein(a) (Lp(a)) concentrations [161,164,166]. Future studies should aim to translate these pro-atherosclerotic risk reduction effects of TCZ to patients without RA. The ASSAIL-IM study, still in phase II, has already demonstrated some data and aims to assess whether TCZ can reduce myocardial damage in patients with ASCVD [81,82].

Aside from quantitative and qualitative changes, TCZ improves endothelial function and decreases oxidative stress, expression of VCAM, and pro-thrombotic status by modulating the pro-thrombotic and pro-inflammatory phenotype of monocytes; it also induces NETosis [167].

The impact of TCZ on arterial stiffness—an independent predictor of cardiovascular risk—has also been evaluated. The results were either conflicting [163] or showed that TCZ reduces pulse wave velocity (PWV) [168,169], while cIMT was not influenced [169]. Regarding traditional cardiovascular risk factors, there were no significant changes in BP [169], but there was a higher prevalence of arterial hypertension among patients treated with TCZ than among those treated with MTX [170]. TCZ also improved the distribution of fat to peripheral tissues and the skeletal muscle mass index [171].

Compared to other bDMARDs, TCZ has a reduced risk of MACE, being superior to abatacept [172] and anti-TNF-α [173], but with no major differences between it and adalimumab or etanercept [173].

Sarilumab, the other monoclonal antibody that binds to the IL-6 receptor, seems to have similar efficacy to TCZ in terms of clinical and radiological improvement of RA [174], while being clinically and functionally superior to adalimumab [175]. The incidence of MACE with sarilumab, whether in combination with csDMARDs or as monotherapy, did not differ from that in patients without DMARDs [176]. Although the changes in lipid profile are the same, studies on the relationship between sarilumab and cardiovascular risk are limited compared to those on TCZ.

### 5.2. Anti-TNF-α

TNF-α, a cytokine produced by activated macrophages and monocytes as well as natural killer (NK) cells, plays a key role in the pathogenesis of RA, due to its pro-inflammatory effects. It is also involved in defending organisms against infection, bone remodeling, and cancer. Increased endothelial permeability to circulating blood cells, NO reduction, ROS production, and the ability to promote dyslipidemia and insulin resistance are mechanisms underlying atheromatous plaque formation [151,177]. It is worth noting that patients with MI who were being monitored for recurrence of MACE showed steadily increased TNF-α levels [178]. Understanding the mechanisms of action of this cytokine has led to the development of targeted therapies such as TNF-α inhibitors. These were the first bDMARDs approved for RA treatment, and all five currently available [177] are described in Table 2.

Data from studies and clinical trials show a reduction in cardiovascular risk in patients treated with TNF-α inhibitors. Comparative studies between anti-TNF-α drugs and csDMARDs demonstrated a 20–30% reduction in cardiovascular risk in the first six months after the introduction of anti-TNF-α drugs [179]. Moreover, anti-TNF-α drugs may reduce the risk of all acute cardiovascular events, but especially of MI or stroke, as suggested by two meta-analyses. [180]. The cardioprotective effect increases proportionally the faster the bDMARDs with anti-TNF-α activity are introduced, but also the longer they are maintained [181]. This is also supported by two other studies in which an increased cardiovascular risk was observed upon [182] and within 6 months of [183] bDMARDs’ discontinuation. In addition to the impact on the occurrence of an acute cardiovascular event such as MI, anti-TNF-α therapy may influence the prognosis of patients after such an event. As regards post-MI mortality, Low et al. [181] demonstrated that patients in whom bDMARDs were stopped 3 months prior to the MI had a threefold higher mortality rate than those receiving anti-TNF-α drugs. No correlation was found between severity or mortality rate and TNF-α inhibitors versus csDMARDs in this study [181]. However, these effects do not apply to all patients, since Ljung et al. [184] showed that patients with low disease activity (as assessed by DAS-28)—referred to as responders—had a 50% lower rate of developing acute coronary syndrome compared to non-responders.

Insights on the effects of anti-TNF-α drugs on the lipid profile have conflicting results. Some studies have reported a significant increase in TC, LDL-C, HDL-C, or ApoA1 and ApoB [185,186], while others have shown no effect on TC and its fractions or triglycerides [187,188] for adalimumab. There was no effect of adalimumab on cholesterol efflux despite inhibiting cholesterol uptake in macrophages [188]. Infliximab, on the other hand, seems to have greater effects on serum lipid levels, with most of the studies proving that it can induce long-lasting increases in TC, LDL-C, HDL-C, and triglycerides [189,190]. In patients treated with golimumab and MTX, increases in TC, LDL-C, and HDL-C were observed compared to those receiving monotherapy with MTX [191], while for certolizumab there are non-specific data available [184,185]. As for etanercept, the ApoB/ApoA ratio was significantly lower in responders among RA patients, while HDL-C increased significantly, with these results demonstrating its favorable effects on the lipid profile [192]. No significant change in LDL-C or triglycerides was reported [192,193].

There is evidence that TNF-α inhibitors have a positive effect on endothelial dysfunction, although this has been observed mainly in patients without many cardiovascular risk factors [194]. Improvements have also been seen in NO bioavailability and ROS production in patients treated with both infliximab and MTX [195]. Low levels of SAA [186] and ADMA [196], along with reduced levels of inflammatory markers such as CRP, phospholipase A2, or fibrinogen, further help to improve cardiovascular risk.

Effects of anti-TNF-α therapy on arterial stiffness showed significant reduction for adalimumab, etanercept, and infliximab after 8–56 weeks of follow-up, independent of other factors such as clinical response or age. The impact appeared to be more pronounced for the first two than for infliximab [197,198,199]. There were no effects reported on cIMT [168,186,198], except for one study showing that anti-TNF-α therapy may be effective in slowing the progression of cIMT, but this is dependent on the long-term disease [200]. As for the impact on traditional cardiovascular risk factors, insulin resistance appeared to improve with infliximab therapy [189]. Although there is evidence that patients receiving TNF-α inhibitors present a risk of developing arterial hypertension [201], many studies do not show a direct correlation between them [187,190]. Nevertheless, monitoring BP during bDMARDs should be part of the therapeutic management.

Regarding the risk of developing acute cardiovascular events, there is evidence showing that the risk for the occurrence of MACE is lower in patients treated with etanercept compared to TCZ [202] or tofacitinib (a janus kinase inhibitor) [203], while another study found no difference in the risk of MACE between patients treated with tofacitinib and with adalimumab [204].

### 5.3. Anti-CD20

In addition to previously described mechanisms involved in the pathogenesis of atherosclerosis, B-cell activation plays an important role by stimulating Th1, with a pro-atherogenic effect, and inhibiting IL-17, with an anti-atherogenic effect [205]. Both B1 (by producing IgM antibodies) and B2 have been shown to promote atherosclerosis [206]. Moreover, B cells stimulate the production of different cytokines, such as IL-6, IL-8, IL-10, and TNF-α [207]. Finally, anti-CD20 treatment, through the consumption of B2 cells, slows the progression of atherosclerosis [208]. There is some evidence demonstrating the potential anti-atherogenic role of anti-CD20 treatment. Treatment with anti-CD20 drugs in mice led to decreased infarct area and improved cardiac remodeling [209].

Rituximab (RTX), a monoclonal CD20 antibody, works by depleting B2 cells. It has been shown to be effective in the treatment of RA, by improving clinical symptoms and slowing disease progression. RTX is a second-line biologic agent, used in case of therapeutic failure of another bDMARD [152].

Significant increases in HDL-C along with decreases in the ApoB/ApoA1 ratio (seen as an atherogenic index) have been reported, while TC and triglycerides were increased in two studies [210,211]. However, other studies showed no changes in HDL-C or triglycerides, along with significant increases in TC and LDL-C [169,210]. Due to conflicting findings, further studies are needed to elucidate the impact of RTX on lipid profiles.

There was no significant effect on B, or on PWV, as demonstrated in three studies [169,212,213], although in one study an improvement in cIMT was observed [213].

Improved cardiovascular risk in patients treated with RTX may also result from reduced inflammatory status, with studies showing decreases in CRP, VSH, DAS-28 [211], and SAA [210], as well as enhanced endothelial function [214].

According to the literature, the reduction in the risk of acute cardiovascular events such as MIs using RTX is similar to that from using anti-TNF-α drugs [215].

### 5.4. Anti-CD80/86

As described in Section 3, T cells play a pivotal role in the immune response in native atherosclerosis. Abatacept (whose mechanism of action is described in Table 2) shows strong clinical promise for cardiovascular risk prevention, since T-cell CD28-CD80/86 co-stimulation is essential for accelerated atherosclerosis [216].

Assessing factors that influence cardiovascular risk, studies have not reported changes in TC, triglycerides, LDL-C, or HDL-C levels for abatacept [169,217]. There is one study showing an increase in LDL-C [218], while there two showing improvements in HDL-C, both quantitatively and qualitatively [218,219]. No significant modification in cIMT [169,220] or BP [169] was observed, while BMI showed an upward trend; however, insulin sensitivity appeared to be improved [221].

Compared to other bDMARDs—specifically, to TNF-α inhibitors—abatacept demonstrated better cardioprotective effects [172,222]. Jin et al., in their review [220], noted that patients treated with abatacept had a 28% lower risk of MACE compared with anti-TNF-α therapy and a 36% increased risk of MACE compared with those starting TCZ; nevertheless, only the composite outcome showed this effect of TCZ. In another study, this characteristic was only found in patients with diabetes mellitus [223].

## 6. Conclusions

Cardiovascular risk is significantly increased in RA patients, as shown by a meta-analysis of 17 studies including 124,894 RA patients, which confirmed the increased risk of MI or stroke [224]. EULAR recommends that in patients with a disease duration of more than 10 years, positive RF and/or ACPA, and extra-articular manifestations, the cardiovascular risk should be multiplied by 1.5-fold. Increased cardiovascular risk in these patients cannot be explained by the presence of traditional cardiovascular risk factors alone. RA has been shown to be an independent risk factor for CVD, and increased inflammatory status leads to accelerated atherosclerosis.

In addition to disease control, optimal management of RA patients also requires control of inflammation and cardiovascular risk factors. Since RA patients’ risk of MI is 70% higher, and sudden death is more common among them than in the general population, atherosclerosis should be the target of therapies aimed not only at achieving remission, but also at reducing cardiovascular risk. Cardiovascular risk reduction in this group of patients is still an unmet need, although both favorable and adverse effects of the widely used therapies are known. Early initiation of biological therapy, with longer and continuous use, has been shown to reduce cardiovascular morbidity and mortality in RA patients. However, whether biological therapy exerts cardioprotective and anti-atherosclerotic effects beyond reducing inflammation remains to be demonstrated. The impact of different bDMARDs on BP control, metabolic syndrome or BMI, endothelial function, and arterial stiffness or atherosclerotic plaques is uncertain and opens up new research perspectives.

In conclusion, further studies are needed in order to detect the subgroup of RA patients requiring additional CVD screening and/or aggressive primary prevention. Future prospective clinical trials are warranted in order to identify accessible biomarkers that can predict ASCVD in RA patients. Moreover, studies leading to the implementation of valid, easy-to-perform and -interpret risk scores in cardiovascular risk assessment are required.

The key points of this article are as follows:

☐ RA patients are complex patients requiring a multidisciplinary approach, especially because the interaction between traditional cardiovascular risk factors and disease-specific inflammation increases cardiovascular risk.
☐ RA patient management involves the following:
  ■ Caution when prescribing medication that contributes to increased cardiovascular risk (e.g., COX-2 inhibitors, glucocorticoids, leflunomide);
  ■ Management of cardiovascular risk factors (i.e., antihypertensive and hypolipidemic treatment should be administered according to current guidelines);
  ■ Induction of disease remission and optimal control of systemic inflammation;
  ■ Quantifying cardiovascular risk and detecting early atherosclerosis;
  ■ Early implementation of targeted bDMARDs in selected patients.

## Figures and Tables

**Table 2:** Main bDMARDs used in RA treatment, their mechanisms of action, and their effects.

Classes of bDMARDs	bDMARDs	Biosimilar	Mechanism	Effects
**Anti-IL-6**	***Tocilizumab***	-	Monoclonal antibodies act as IL-6 receptor antagonists, to which they bind and prevent this cytokine from being fixed at this level	Clinical and biological improvement, slowing or stopping disease progression; preventing joint destruction Increased efficiency as a therapy in RA patients [150,152,155,158]
	***Sarilumab***	-		
**Anti-TNF-α**	***Infliximab***	**√**	Chimeric IgG1k monoclonal antibody	Neutralization of biological effects of TNF-α, such as stimulation of synthesis and release of pro-inflammatory cytokines, prostaglandins, and platelet-activating factors; endothelial dysfunction; development and progression of atheromatous plaques; cardiac remodeling [150,152]
	***Adalimumab***	**√**	Human IgG1 monoclonal antibody	
	***Golimumab***	-	Fully human monoclonal antibody	
	***Certolizumab pegol***	-	PEGylated monoclonal antibody formed with a humanized Fab fragment	
	***Etanercept***	**√**	Soluble TNF-α receptor	
**Anti-CD20 (anti-LB)**	***Rituximab***	**√**	Chimeric monoclonal anti-CD20 antibody; the antigen CD20 is expressed on the surface of LB	Induces B2 cell depletion Clinical improvement, slowing or stopping disease progression; preventing joint destruction Increased efficacy in combination with MTX [150,152,155]
**Anti-CD80/86 (anti-LT)**	***Abatacept***	-	Soluble receptor consisting of a fusion molecule that blocks the binding of CD80 and CD86 receptors on the antigen-presenting cell (APC), thereby inhibiting T-cell activation	Clinical improvement, slowing disease progression; preventing joint destruction Therapeutic effects and safety profile similar to adalimumab [150,152,155]

## Data Availability

Not applicable.
